# LocTree2 predicts localization for all domains of life

**DOI:** 10.1093/bioinformatics/bts390

**Published:** 2012-09-03

**Authors:** Tatyana Goldberg, Tobias Hamp, Burkhard Rost

**Affiliations:** ^1^TUM, Bioinformatik-I12, Informatik, Boltzmannstrasse 3, Garching 85748, Germany; ^2^New York Consortium on Membrane Protein Structure (NYCOMPS) and Department of Biochemistry and Molecular Biophysics, Columbia University, New York, NY 10032, USA

## Abstract

**Motivation:** Subcellular localization is one aspect of protein function. Despite advances in high-throughput imaging, localization maps remain incomplete. Several methods accurately predict localization, but many challenges remain to be tackled.

**Results:** In this study, we introduced a framework to predict localization in life's three domains, including globular and membrane proteins (3 classes for archaea; 6 for bacteria and 18 for eukaryota). The resulting method, LocTree2, works well even for protein fragments. It uses a hierarchical system of support vector machines that imitates the cascading mechanism of cellular sorting. The method reaches high levels of sustained performance (eukaryota: Q18=65%, bacteria: Q6=84%). LocTree2 also accurately distinguishes membrane and non-membrane proteins. In our hands, it compared favorably with top methods when tested on new data.

**Availability:** Online through PredictProtein (predictprotein.org); as standalone version at http://www.rostlab.org/services/loctree2.

**Contact:** localization@rostlab.org

**Supplementary Information:** Supplementary data are available at *Bioinformatics* online.

## 1 INTRODUCTION

### 1.1 Localization related to function

Archaea, bacteria and eukaryota form the three domains of life (Woese *et al*., 1990). Archaea and bacteria are prokaryotes, i.e. organisms that lack a nucleus and other membrane-bound organelles. Prokaryotic cells surround a single compartment by the plasma membrane (Gram-negative bacteria add an outer membrane). Eukaryotic cells are organized into several membrane-bound compartments. Subcellular localization is one aspect of cellular function as exemplified in the cellular component in the gene ontology (GO, Ashburner *et al*., 2000). Proteins contributing to the same physiological function often co-localize (Andrade *et al*., 1998; Jensen *et al*., 2002; Rost *et al*., 2003). Although proteins can be functional in different compartments (e.g. *importins* that shuttle other proteins into the nucleus), most proteins of known function complete their tasks as ‘natives’ of one particular compartment. For instance, many nuclear proteins are imported into the nucleus without being re-exported (Cokol *et al*., 2000); virulence-associated proteins are likely to be secreted in many bacterial pathogens (Durand *et al*., 2009). Increasing evidence suggests that proteins form temporary complexes to act in concert, resembling a macromolecular just-in-time production facility (Farhah Assaad TUM-WZW, personal communication). The knowledge of localization may, therefore, be important to understand protein interactions and cellular mechanisms.

### 1.2 Better annotations of function by predicting localization

The sequence-annotation gap refers to the gap between the number of proteins with known sequences and with comprehensive functional annotations. Next-generation sequencing explodes this gap despite increasing high-throughput experiments. Reliable automated predictions of protein function could counter this trend (Al-Shahib *et al*., 2007; Bairoch and Apweiler, 2000). Subcellular localization is one objective and easily definable aspect of function; many *in silico* prediction methods have been developed:

1. Sorting signals: Sorting signals (short motifs recognized by shuttle proteins) provide ‘biologically meaningful’ explanations for particular predictions. Most localization signals remain experimentally elusive (Nair and Rost, 2005) and many of the known signals have little coverage, i.e. allow the identification of very few proteins known to localize to that compartment (Wrzeszczynski and Rost, 2004). In addition, some proteins are sorted non-classically—not signal peptide triggered (Bendtsen *et al*., 2004).
2. Homology-based inference: The best localization predictions use annotations from close homologs (Nair and Rost, 2002b). This technique has limited reach because reliable inference requires high sequence similarity. It also has accuracy limitations: two 500-residue proteins may be sorted differently due to a 5-residue motif.
3. Text-based analyses: Text analysis-based methods infer localization from experimental information contained in the literature, such as PubMed abstracts (Brady and Shatkay, 2008) or from controlled vocabularies of curated databases, such as SWISS-PROT keywords (Nair and Rost, 2002a). All text-based methods are restricted in coverage as they rely on existing annotations.
4. *De novo: De novo* methods predict localization without requiring significant sequence similarity to annotated proteins. These methods are solely amino acid composition based (Chou, 2001; Park and Kanehisa, 2003; Reinhard and Hubbard, 1998).
5. Hybrid approaches combine several of these original four concepts (Blum *et al*., 2009; Briesemeister *et al*., 2009; Hoglund *et al*., 2006; Horton *et al*., 2007; Nair and Rost, 2005).

Here, we present a novel sequence-based method for predicting the subcellular localization of all proteins in all domains of life. Our method addresses several shortcomings of existing approaches. (i) We provide a common framework for all domains of life and this framework is more robust with respect to sequencing mistakes than other methods. (ii) We increase the number of classes covered by a single consistent framework: 3 localization classes for archaea, 6 for bacteria and 18 for eukaryota. Predictions distinguish between integral trans-membrane and water-soluble globular (non-membrane) proteins. (iii) Similar to LocTree (Nair and Rost, 2005), we implemented a decision tree-like architecture of localization classes imitating the cellular protein sorting mechanisms. A tree-like structure accommodates the similarity of sorting signals specific to similar compartments (Alberts *et al*., 2007; Rusch and Kendall, 1995). (iv) We provide scores for the reliability of a prediction; these are crucial because they allow focusing on the most relevant results. All the above advantages were achieved without sacrificing performance. In our hands, LocTree2 performed significantly better than other methods on nearly all data sets tested.

## 2 METHODS

### 2.1 Data sets for development and evaluation

We extracted protein sequences with explicit annotations of subcellular localization from SWISS-PROT release 2011_04 (Bairoch and Apweiler, 2000). Excluded were annotations based on non-experimental findings (‘potential’, ‘probable’ or ‘by similarity’). Also excluded were proteins with multiple or ambiguous localization annotations (e.g. Gram-negative proteins annotated with ‘cell membrane’ could be in the inner or outer membrane). Proteins lacking the term ‘membrane’ were considered as ‘non-membrane’. Transmembrane proteins, i.e. proteins spanning the membrane at least once, were found using terms ‘single-pass’ or ‘multi-pass’. Through the NCBI taxonomy (Benson *et al*., 2010), proteins were assigned to one of the three domains (archaeal, bacterial or eukaryotic). Sequence bias was reduced through UniqueProt (Mika and Rost, 2003), applied independently for archaea, bacteria and eukaryota. This bias-reduction ascertained that no pair of proteins in the final set had BLAST2 (Altschul *et al*., 1990) E-value (EVAL) ⩽10^−3^ or HSSP-value (HVAL) *>* 0 (Rost, 1999; Sander and Schneider, 1991). For alignments longer than 250 residues, HVAL *<* 0 implies that the maximal pairwise sequence identity was 20% (Rost, 1999). Filtering by HVAL and EVAL ensured that homology-based inference would be less accurate than our previous LocTree method (Nair and Rost, 2005). Alignments of fewer than 35 residues were removed, which is roughly the maximal length of known localization signals (Cokol *et al*., 2000). The final sets contained 59 archaeal, 479 bacterial and 1682 eukaryotic proteins (Supplementary Table S1).

### 2.2 Data sets for additional testing

After completing the development, we benchmarked our single best method against publicly available state-of-the-art methods. This involved the following independent test sets: (i) 28 bacterial and (ii) 52 eukaryotic proteins added to SWISS-PROT between releases 2011_04 and 2012_02; (iii) 43 *Arabidopsis thaliana* and (iv) 201 *Homo sapiens* proteins taken from LocDB (Rastogi and Rost, 2011). Proteins with HVAL > 5 to any previously used protein (including those discarded during the redundancy reduction) were excluded. This threshold corresponds to 25% pairwise sequence identity over 250 residues aligned. UniqueProt was used to reduce redundancy between the data sets and within each data set at HVAL > 0 and BLAST2 EVAL ⩽10^−3^ with the minimum alignment length of 35 residues. We never used any of the remaining proteins (Supplementary Table S2) for any further improvement of our method. With the exception of LocTree, which used homology-based and text analysis-based predictions of SWISS-PROT proteins, and WoLF PSORT, which extracted an additional set of *Arabidopsis thaliana* proteins from Gene Ontology (Ashburner *et al*., 2000), the other methods tested here did not use any of the proteins in these independent test sets, as they were trained on data from SWISS-PROT releases before April 2011.

### 2.3 Additional data sets for comparison with LocTree

A question not addressed by the above data sets and comparisons is as follows: to which extent did our method benefit from the growth of the databases since 2005? In a separate analysis, all proteins for which localization had been annotated before 2005 served as training set and all from the above cross-validation set without sequence similarity (HVAL *>* 0 and EVAL ⩽10^−3^) to this training set were used to compare LocTree2 and our previous method LocTree (Supplementary Table S3). No parameter optimization was applied when re-training our new method.

### 2.4 Prediction method

Each domain of life was considered as a separate learning problem yielding three different systems of decision trees (archaea: 3 classes, bacteria: 6 and eukaryota: 18; Fig. 1). Each leaf (rectangles) represents one localization class, and each internal node (circles) is a binary support vector machine (SVM). Most methodological aspects of the new method combine existing ideas. We briefly describe the main aspects here and leave the precise, formal definitions to the Supplementary Sections 1–3.

**Fig. 1.**
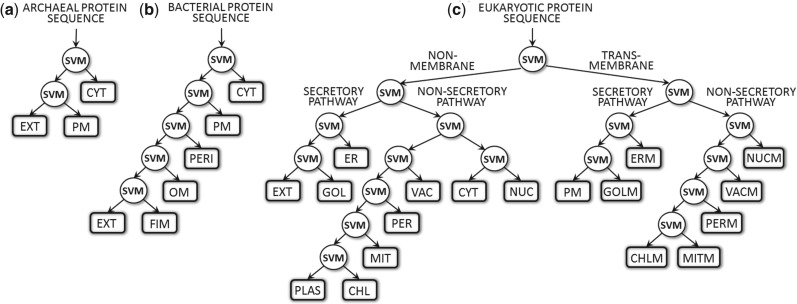
Hierarchical architecture of LocTree2. The localization prediction follows a different tree for each of the three domains of life: **(a)** archaea, **(b)** bacteria and **(c)** eukaryota. Each hierarchy mimics the biological sorting mechanism in that domain (in eukaryotes membrane and non-membrane proteins are treated separately). The branches represent paths of the protein sorting, the leaves the final prediction of one localization class and the internal nodes are the decision points along the path. These decisions are implemented as binary support vector machines (SVMs). CHL, chloroplast; CHLM, chloroplast membrane; CYT, cytosol; ER, endoplasmic reticulum; ERM, endoplasmic reticulum membrane; EXT, extra-cellular; FIM, fimbrium; GOL, Golgi apparatus; GOLM, Golgi apparatus membrane; MIT, mitochondria; MITM, mitochondria membrane; NUC, nucleus; NUCM, nucleus membrane; OM, outer membrane; PERI, periplasmic space; PER, peroxisome; PERM, peroxisome membrane; PM, plasma membrane; PLAS, plastid; VAC, vacuole; VACM, vacuole membrane

#### 2.4.1 Input

For each protein, sequence profiles were created by BLAST-ing (Altschul *et al*., 1997) queries against an 80% non-redundant database combining SWISS-PROT, TrEMBL (Bairoch and Apweiler, 2000) and the Protein Data Bank (Berman *et al*., 2000). Our method only used information available through these profiles.

#### 2.4.2 Profile kernel

Kernel methods (such as the SVM) differentiate between the input and the feature space. Here, the input space was spanned by all possible sequence-profile tuples. The feature space was implicitly given by the profile kernel (Kuang *et al*., 2004) that maps such a tuple to a vector indexed by all possible subsequences of length *k* from the alphabet of amino acids. Each element represents one particular *k*-mer and gives the number of identical *k*-mers with a score below a user-defined threshold *s*. This score is calculated as the ungapped cumulative substitution score in the corresponding sequence profile. We can then define the profile kernel function as the dot product between the two *k*-mer vectors of the two sequence-profile tuples. Essentially, the method identifies stretches of *k* adjacent residues in the query that are most informative for the prediction of localization and then matches these in query protein.

#### 2.4.3 SVM training

SVMs were trained using a pre-computed kernel matrix of all training proteins. For the profile kernel, the matrix can be calculated very efficiently with the suffix tree-based ‘kernel trick’ introduced by the groups of Christina Leslie and Bill Noble (Leslie *et al*., 2004). We found other string kernels (Leslie *et al*., 2004; Lodhi *et al*., 2002) either slower in runtime or worse in performance (Supplementary Table S4). The SVM was implemented by the WEKA (Holmes *et al*., 1994) sequential minimal optimization (Platt, 1998). Platt Scaling (Platt, 1999) mapped the raw SVM score of the predicted class into a reliability between 0.5 and 1.0.

#### 2.4.4 Tree-like hierarchy of SVMs

The tree model (Fig. 1) was built by training binary SVM classifiers; each of those was trained on different sets of proteins. To this end, we first looked at one of the two child nodes of an internal node (e.g. internal node: root and child node: non-cytoplasmic; Fig. 1a) and collected all the training proteins of its leaf classes (e.g. EXT and PM; Fig. 1a). They were assigned to class A. Then we did the same for the second child node (e.g. CYT) and assigned its proteins to class B. Now, we could train the SVM of the parent node with the proteins in classes A and B. Repeating this for all internal nodes, we trained the entire tree model.

#### 2.4.5 Reliability index

The reliability of the predicted class (leaf node) for a sequence-profile tuple was compiled as the product over the reliabilities of all parent nodes (as described in [Reinhardt and Hubbard, 1998]). We formed the LocTree2 reliability index (RI) by multiplying an integer of this value by 100. As the prediction confidence did not change for scores *<*20, the index was re-normalized accordingly.

### 2.5 Cross-validation

For training and testing, stratified 5-fold cross-validation was performed with each of the three sequence unique development data sets described before. This required several additional cross-validation layers to optimize various free SVM and multi-class learning parameters (Supplementary Section 1 for details). Note that we never used any information of the test split during a training phase. Entire rounds of cross-validation yielded comparisons to other multi-class learners (e.g. ENDs [Frank and Kramer, 2004]). Additionally, the influence of redundancy reduction was monitored; this suggested a controlled addition of redundancy after an initial reduction to be favorable (Supplementary Section 4).

### 2.6 Performance evaluation

Looking at predictions from the perspective of a single localization class *L* suggests various performance measures: the accuracy is the ratio between the number of correctly predicted proteins in localization *L* and all proteins predicted to be in *L*. Coverage is the ratio ‘correctly predicted in *L*/all proteins observed in *L′*. Both values are combined in the geometric average gAv. The overall accuracy *Q*(*n*) as the number of correctly predicted proteins across *n* classes divided by the number of observed proteins in these classes provides the perspective across all classes. Standard error for all measurements was estimated over 1000 bootstrap sets; i.e. randomly select *n* proteins without replacement from the original data set (in our experience, bootstrapping without replacement typically yields error estimates that are more conservative/long lived than those with replacement). For each bootstrapped set, the performance *x_i_* is estimated (e.g. accuracy). These 1000 estimates provided the standard deviation of *x_i_* with the typical standard error = standard deviation divided by 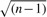 , where *n* is the number of bootstrapped sets.

### 2.7 State-of-the-art prediction methods

We compared LocTree2 with the following publicly available state-of-the-art methods using default parameters.

*CELLO* 2.5 (Yu *et al*., 2006) is a system of SVMs that predicts localization of bacterial proteins to 5 classes and eukaryotic proteins to 12 classes. Predictions are based on sequence-derived features.
*LocTree* (Nair and Rost, 2005) predicts localization of non-membrane proteins from prokaryotes (three classes) and eukaryotes (six classes for plants and five for others) through the hierarchy of binary SVMs. The method uses features representing the entire protein and N-terminus specifics.
*MultiLoc*2 (Blum *et al*., 2009) uses SVMs that integrate sequence-based features with phylogenetic profiles and GO terms. It predicts 9 localization classes for animals/fungi and 10 plant classes (adding in chloroplast).
*PolyPhobius* (Kall *et al*., 2005) uses a hidden Markov model (HMM) for the prediction of transmembrane protein topology and signal peptides. It incorporates homology information for the increased prediction accuracy.
*PSORTb* 3.0 (Yu *et al*., 2010) predicts four classes for archaea/Gram-positives and five for Gram-negatives. It combines several classifiers by a Bayesian network to generate a final prediction of localization.
*Scampi* (Bernsel *et al*., 2008) predicts transmembrane protein topology through an HMM. Predictions are based on the experimental scale of position-specific amino acid contributions to the free energy of membrane insertion coupled with the positive-inside rule.
*WoLF PSORT* (Horton *et al*., 2007) is a *k*-nearest neighbor classifier that predicts 12 localization classes for eukaryotes from sequence-based features. Similar to its predecessors (PSORT), it uses a tree hierarchy resembling cellular sorting and a battery of established prediction methods.

Unlike all others, *PolyPhobius* and *Scampi* do not aim at predicting localization. Instead, they focus on the prediction of which residues are inserted as transmembrane helices into the lipid bilayer. In the context herein, those two methods are compared to demonstrate that LocTree2 could even stand up to specialists that optimize the distinction of membrane and non-membrane proteins in their own domain of specialization.

## 3 RESULTS AND DISCUSSION

### 3.1 Three prediction trees for three domains of life

Our first hierarchal method, LocTree (Nair and Rost, 2005), used a concept initially introduced by the work on PSORT carried by Paul Horton and initiated by Kenta Nakai and Minoru Kanehisa (Horton *et al*., 2007; Nakai and Horton, 1999; Nakai and Kanehisa, 1991). For LocTree2, many alternative trees were tested. Trees mimicking the cellular protein trafficking using binary models at the internal nodes (Fig. 1) were similar in performance but much faster than other multi-class schemes, for example ENDs (Frank and Kramer, 2004) (Supplementary Table S5). Starting at the root classifier (e.g. non-membrane/trans-membrane; Fig. 1c), the decisions at each node are followed until reaching a leaf (e.g. mitochondria membrane [MITM]). This leaf corresponds to the predicted localization class (development set in Supplementary Table S1).

### 3.2 Cross-validated Q18 = 65% for eukaryotes

**Fig. 2.**
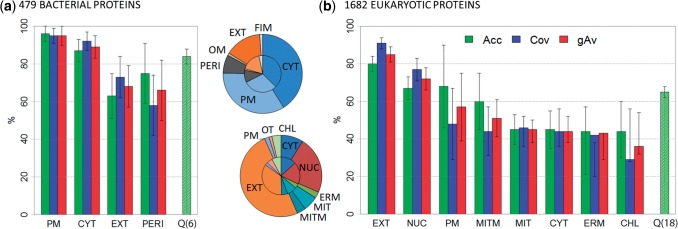
High performance in cross-validation. For the cross-validation sets (**a**: averages over 479 bacterial proteins and **b**: averages over 1682 eukaryotic proteins), LocTree2 reached high levels of sustained performance. Overall, performance tended to correlate with the number of representatives (pie charts: inner ring: composition in the corresponding data set and outer ring: composition in correct predictions). Exceptions were membrane bound classes in eukaryotes for which the performance tended to be better than that for the corresponding non-membrane bound class (e.g. MIT = mitochondrial proteins versus MITM = membrane-linked mitochondrial proteins). Localization classes as in Figure 1; performance measures: Acc, accuracy; Cov, coverage; gAv, geometric coverage of Acc and Cov; *Q*, overall prediction accuracy (Q6 for six and Q18 for 18 classes). Standard errors were estimated by bootstrapping (see Section 2). Classes with less than 20 members were excluded

The first decision for eukaryotic proteins was: does it have an integral transmembrane region or not (Fig. 1c). This decision was correct for over 90% of all proteins (Supplementary Figure S1b). Both membrane and non-membrane proteins were further classified into ‘secreted’ and ‘not secreted’; this decision reached Q4 = 83% accuracy (Q4 = four state accuracy, see Section 2 for definition of *Qn*; Supplementary Figure S1b). Descending the tree toward the leaves that represent the final predictions, the distinction between intra-cellular and secretory pathway into 10 classes for non-membrane and 8 classes for transmembrane proteins was less accurate (Q8 = 75%; Supplementary Figure S1b). The class with most observations (extra-cellular: 35% of data) was also predicted best (accuracy: 80%, coverage: 91%, Fig. 2b, Supplementary Table S6) followed by nuclear proteins (accuracy: 67%, coverage: 72%). The overall accuracy for 18 classes Q18 reached 65% (18-state accuracy, Fig. 2b).

Overall, performance correlated with the amount of available experimental information (Fig. 2b: inner and outer pies very similar), with the important exception that membrane-bound proteins tended to be predicted more accurately than their corresponding non-membrane bound neighbors (e.g. mitochondria [MIT] versus MITM in Fig. 2b).

### 3.3 Highest numerical performance for prokaryotes

LocTree2 performed very well in the cross-validation of archaea (three classes) with overall levels of accuracy and coverage numerically suggested to reach 100% (Supplementary Table S7). These numbers most likely over-estimate performance due to the limited data. For bacteria (six classes), the overall accuracy was 84% (Fig. 2a); the most accurate sub-classification was the sorting into plasma membrane (accuracy: 96%, Fig. 2a, Supplementary Table S6) followed by cytosol (accuracy: 87%).

### 3.4 Performance best for more reliably predicted proteins

**Fig. 3.**
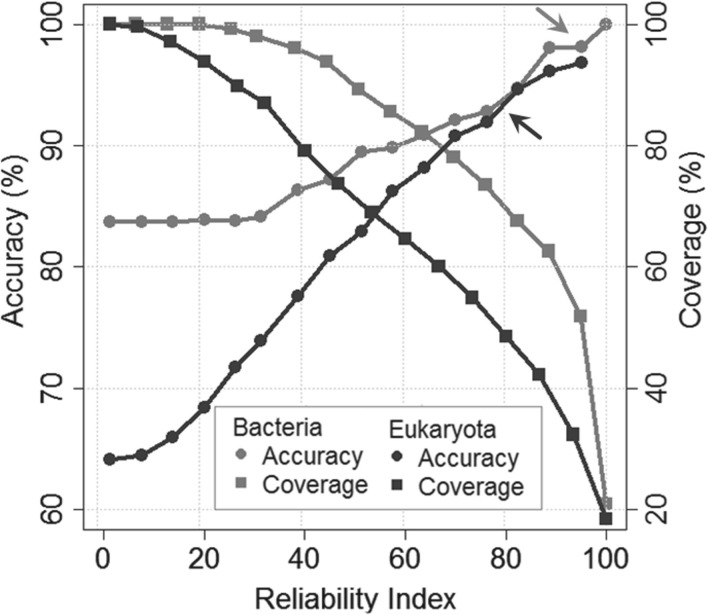
More reliable predictions better. The curves show the percentage accuracy/coverage for LocTree2 predictions above a given threshold in the reliability index (from 0 = unreliable to 100 = most reliable). True positives are the number of correct predictions with reliability indices above the given threshold, false negatives are the number of correct predictions with reliability indices below the threshold and false positives are the number of wrong predictions with reliability indices above the threshold. The curves were obtained on cross-validated test sets of bacterial (gray line) and eukaryotic (black line) proteins. Half of all eukaryotic proteins are predicted at RI*>*80; for these, Q18 is above 92% (black arrow). As the number of localization classes is lower for bacteria, the corresponding number in accuracy is higher (Q6 is above 95% at 50% coverage, gray arrow)

One way to focus on more reliable predictions is to compile a consensus for alternative methods. Often, method internal reliability indices are far superior at spotting the best predictions than combinations of different methods (Eyrich *et al*., 2003). LocTree2 computed the reliability index (RI) as the joint probability over all individual SVM scores (see Section 2, Fig. 3). For instance, the 50% of the proteins with highest reliability reached levels of overall accuracy Q6 = 98% for bacteria (Fig. 3, gray arrow) and Q18 = 92% for eukaryota (Fig. 3, black arrow). To pick another point, almost 40% of all eukaryotic proteins were predicted at RI *>* 85; for these, Q18 was above 95%. Thus, two in the top 40 predictions in 100 were wrong in one of 18 states (e.g. nuclear instead of nuclear membrane).

### 3.5 LocTree2 competitive for new proteins

There is no value in comparing LocTree2 with other methods based on values for performance published because of the differences in, for example data sets and cross-validation setups. Comparisons based on running other methods on our data are also problematic due to possible overlap in training and due to possible performance over-estimates of our own method. The only meaningful way is to use proteins that are non-redundant with respect to each other and with respect to any protein used for the development of the methods tested. Toward this end, we collected the most recently added annotations in SWISS-PROT. The price for this ‘clean’ comparison was the tiny data set: 28 bacterial and 52 eukaryotic proteins after redundancy reduction (explaining high standard errors in Table 1).

**Table 1.** Performance comparison on independent data sets

Method	New SWISS-PROT					LocDB					
	*Bacteria (28)*		*Eukaryota (52)*			*A. thaliana (43)*			*H. sapiens (201)*		
Q(5)	Q(3)	Q(9)	Q(6)	Q(5)	Q(9)	Q(8)	Q(6)	Q(8)	Q(7)	Q(6)
LocTree2	**86** ± **16**	**86** ± **18**	**65** ± **14**	**66** ± **16**	**66** ± **15**	**37** ± **18**	**44** ± **21**	**49** ± **20**	42 ± 8	**44** ± **9**	**51** ± **9**
CELLO v. 2.5	57 ± 22	—	46 ± 16	—	—	26 ± 18	—	—	40 ± 8	—	—
WoLF PSORT	—	—	62 ± 14	—	—	19 ± 15	—	—	**45 ± 8**	—	—
PSORTb 3.0	71 ± 21	—	—	—	—	—	—	—	—	—	—
MultiLoc2	—	—	—	60 ± 16	—	—	24 ± 18	—	—	42 ± 9	—
LocTree		77 ± 21	—	—	62 ± 17	—	—	24 ± 18	—	—	48 ± 9

Data ‘New SWISS-PROT’: 28 sequence-unique bacterial and 52 eukaryotic proteins added to SWISS-PROT between releases 2011_04 and 2012_02 (sequence uniqueness was ascertained both within this set and from any protein in this set to any other protein previously in SWISS-PROT). *Data ‘A. thaliana’ and ‘H. sapiens’*: 43 *Arabidopsis thaliana* and 201 *Homo sapiens* proteins from the LocDB database (as for ‘New SWISS-PROT’: sequence unique with respect to itself and to SWISS-PROT 2011_04). *Qn*, the overall prediction accuracy in *n* classes; highest value in each column in bold; values ± standard error (see Section 2).

CELLO 2.5 and PSORTb 3.0 classified bacterial proteins into five classes and LocTree into three. This was accounted for by grouping bacterial extra-cellular and fimbrium proteins into one common class for predictions using these external methods. We separated Gram-positive from Gram-negative bacterial proteins according to Yu *et al*. (2010) for a comparison with PSORTb 3.0.

Eukaryotic proteins were classified into twelve classes for CELLO 2.5 and WoLF PSORT, into ten classes for MultiLoc2 and into six classes for LocTree. We excluded vacuolar proteins for MultiLoc2 and plasma membrane proteins for LocTree (thereby providing over-optimistic upper performance levels for those methods). WoLF PSORT may predict multiple localizations, and we always took the right one for performance estimates (it was verified that this did not impact estimates significantly). WoLF PSORT and CELLO 2.5 distinguish cytoskeleton and cytoplasm; here, both were considered as cytoplasmic. Another issue was that other methods do not distinguish membrane from non-membrane proteins. Thus, we merged these two classes, i.e. treated nuclear and nuclear-membrane proteins identically, although this approach implicitly sacrificed one of the important strengths of our new method, namely the distinction of these.

The ‘New SWISS-PROT’ bacterial and eukaryotic sets were too small to clearly identify the top performing method given the standard error. However, LocTree2 compared favorably to other state-of-the-art methods (Table 1). Performance estimates for the newly annotated proteins tended to be lower than the values published (except for LocTree and MultiLoc2). For LocTree2, the overall accuracy was similar for the cross-validation experiment (84% ± 4% for bacteria and 65% ± 3% for eukaryota; Fig. 2, Supplementary Table S6) and for the new proteins (86% ± 16% for bacteria and 65% ± 14% for eukaryota; Table 1).

### 3.6 LocTree2 would already have performed well in 2005

Another way to compare two prediction methods is to train and test on the same data set. We trained a version of LocTree2 on proteins for which localization was known when LocTree was trained and tested both on proteins from our newer cross-validation set without sequence similarity to the training set (see Section 2 and Supplementary Table S3). LocTree2 outperformed LocTree reaching levels of overall accuracy Q3 = 80% ± 13% for bacteria and Q6 = 61% ± 8% for eukaryota (LocTree: Q3 = 62% ± 18% and Q6 = 54% ± 8%). Thus, the improvement of LocTree2 originated mainly from the underlying method advancement. LocTree2 trained on the 2011 data reached Q6 = 62% ± 8% and Q18 = 60% ± 9% which is within the standard error of what was obtained on the full cross-validation set (Supplementary Table S6).

### 3.7 High-throughput data ambiguous?

LocDB collects localization annotations mostly from high-throughput experiments; it provided two data sets for the comparison of methods: one for the plant *Arabidopsis thaliana* and the other for *Homo sapiens*. Both sets were redundancy reduced, with respect to each other and with respect to SWISS-PROT version 2011_04. For all the LocDB proteins, all methods appeared to perform substantially worse than for the already ‘tough’ set of newly annotated SWISS-PROT proteins. For the plant, LocTree2 outperformed others (Table 1). Not so for human: WoLF PSORT reached Q8 = 45% ± 8% (versus LocTree2 Q8 = 42% ± 8%). One-third of the correct predictions from WoLF PSORT were for cytoplasmic proteins, which was overall, the most populated class for human proteins in LocDB (Supplementary Table S2).

How to interpret the data from LocDB? As most annotations in LocDB originate from high-throughput experiments, it is very likely that LocDB contains proportionally more errors than SWISS-PROT. All methods by far outperformed random, implying that for random annotation mistakes they would appear to be mostly wrong. Thus, the higher error rate in LocDB might explain why all methods perform worse for the LocDB than for the SWISS-PROT data. Put differently, for a task with over six classes and the given number of proteins, a few mistakes can reduce the average considerably. On the other hand, we might also suspect that high-throughput experiments discover a reality invisible to traditional experimental methods and some of those invisible facts might reveal new sorting mechanisms. Such hidden mechanisms might or might not be ‘discovered’ by prediction methods. If not, those would explain many incorrect predictions. Supposedly, most experts would be very surprised if the second argument (new mechanism) dominated over the first (annotation mistakes of high-throughput experiments). Most likely there is a little bit of both, but we have no means of gauging the relative proportions. Zooming into annotations with several evidences brought the numbers closer, i.e. ‘increased’ the performance, but this was achieved at raising the standard errors to meaningless values (Supplementary Table S8).

We illustrate the situation for a few extreme predictions. (i) ‘Transmembrane emp24 domain-containing protein 3’ (SWISS-PROT TMED3_HUMAN) is annotated as Golgi apparatus by LocDB; LocTree2 maps it to the endoplasmic reticulum (ER) membrane with extremely high reliability (RI=99). This protein belongs to a family of p24 membrane proteins localizing to the ER and to the Golgi complex (Jenne *et al*., 2002). Thus, both LocTree2 and LocDB annotations are correct. (ii) ‘Protein canopy homolog 2’ (CNPY2_HUMAN) is annotated as cytoplasmic in LocDB; LocTree2 predicts ER (RI=73). We found experimental evidence for localization to the ER in HeLa cells (Hirate and Okamoto, 2006). In this case, LocTree2 is correct and LocDB is not. (iii) ‘Methylosome subunit pICln’ (ICLN_HUMAN) is classified as plasma membrane in LocDB, whereas LocTree2 predicts nuclear (RI=55). We could not find any additional information for this case in PubMed, but the protein localization annotation in SWISS-PROT is nuclear. (iv) ‘COMM domain-containing protein 1’(COMD1_HUMAN) is classified as secreted in LocDB, whereas LocTree2 predicts nuclear (RI=50). Again, closer inspection revealed experimental evidence for this protein to be nuclear (Burstein *et al*., 2005).

It remained unclear what to conclude from the above examples. The predictions judged as incorrect by LocDB but having very high reliability scores indicate that the low performance inverts the real picture: rather the annotations are wrong or ambiguous than the strong predictions. For a set of weakest predictions, we observed the opposite. For example (i) ‘Stress-associated endoplasmic reticulum protein 1’ (SERP1_HUMAN) is annotated as ER correctly in LocDB, but LocTree2 maps it to mitochondria with very low reliability (RI = 6). (ii) ‘Spermatogenesis-associated protein 19, mitochondrial’ (SPT19_HUMAN) is classified as mitochondrial correctly in LocDB again, whereas LocTree2 predicts nuclear (RI = 13). A more detailed analysis might succeed in quantifying to which extent the consistent drop in performance for the LocDB data sets reveals more about problems of high-throughput experiments than of *mega-throughput* computations.

### 3.8 Accurate distinction between membrane and non-membrane

As reported before, the SVM that distinguishes between non-/transmembrane proteins in eukaryotes achieved an overall accuracy of 94% ± 2% (Supplementary Figure S1b). This performance was similar to what PolyPhobius achieved on the same data set (95% ± 1%). PolyPhobius appears to be the best expert method that targets the prediction of integral membrane helices directly (Kloppman E., Reeb J. and Rost B., unpublished data). LocTree2 correctly classified all plasma membrane proteins from archaea (Supplementary Table S7), but the data set was too small to provide meaningful performance estimates. For bacterial proteins, the plasma membrane/non-membrane distinction reached 96% ± 4% accuracy (Fig. 2a, Supplementary Table S6). Scampi, the most accurate method for predicting trans-membrane proteins in prokaryotes (Kloppman E., Reeb J. and Rost B., unpublished) was significantly less accurate (89% ± 3%) for the same data.

### 3.9 Advantage over existing methods for sequencing errors

All prediction methods were also benchmarked on protein fragments as they may result from erroneous assembly or wrong gene predictions common in genome projects (Brent and Guigo, 2004). The latter being a special problem for the detection of N-terminal signals because of the wrong predictions of gene starts common when using gene prediction software. Three different ‘models’ simulated worst-case scenarios (over-estimating sequencing mistakes): cleaving off (i) 30 N-terminal residues for all proteins, (ii) 30 C-terminal residues and (iii) randomly picking positions to cleave one third of the sequence. The least ‘damage’was done for the C-term cleavage with LocTree2's accuracy dropping to 60% ± 2% (Supplementary Table S9), which was still within the standard error of what was obtained using the full-length sequences. For other prediction methods, performance dropped much more. Our method also significantly outperformed its competitors on the N-term cleaved sequences and on the sequences with randomly cleaved fragments, reaching the levels above 53% ± 2% accuracy (Supplementary Table S9). This is still accurate enough to provide reliable first estimates of localization for genomic sequences.

## 4 CONCLUSION

The method introduced here, LocTree2, predicts protein subcellular localization through a consistent new framework that ignores many of the relevant features needed for the success of previous methods (such as no predicted aspects of protein structure and function). Nevertheless, it seemed to reach high levels of sustained performance aside from adding new aspects. Among the novel aspects was the large number of 18 localization classes predicted for eukaryota, 6 for bacteria and 3 for archaea. LocTree2 outperformed other methods on almost all data sets tested, implicating an improved ability to capture localization signals in the protein sequence. One example for the success in plucking implicit information is the high precision in the distinction between membrane and globular water-soluble proteins. Our implicit distinction appeared as good as that of the best expert method for predicting integral membrane helices. Another important novelty is the robustness of the method against sequencing errors and its success when applied to protein fragments. This is particularly important in light of high-throughput sequencing, of analyzing ancient DNA with short reads and of the fact that almost 80% of all proteins have multiple domains. This power along with the overall improvement in performance may recommend this new tool as an ideal starting point for comparing the proteomes between organisms and for using localization predictions to aid the prediction of protein function. We imagine that the framework for the method will prove extendable and that future methods will become better simply by using more experimental data and more sequences.
